# The Molecular Characterization of Genetic Abnormalities in Esophageal Squamous Cell Carcinoma May Foster the Development of Targeted Therapies

**DOI:** 10.3390/curroncol30010048

**Published:** 2023-01-03

**Authors:** Ugo Testa, Germana Castelli, Elvira Pelosi

**Affiliations:** Department of Oncology, Istituto Superiore di Sanità, Viale Regina Elena 299, 00161 Rome, Italy

**Keywords:** esophageal cancer, esophageal squamous cell carcinoma, genome sequencing, genetic heterogeneity, targeted therapy

## Abstract

Esophageal cancer is among the most common tumors in the world and is associated with poor outcomes, with a 5-year survival rate of about 10–20%. Two main histological subtypes are observed: esophageal squamous cell carcinoma (ESCC), more frequent among Asian populations, and esophageal adenocarcinoma (EAC), the predominant type in Western populations. The development of molecular analysis techniques has led to the definition of the molecular alterations observed in ESCC, consistently differing from those observed in EAC. The genetic alterations observed are complex and heterogeneous and involve gene mutations, gene deletions and gene amplifications. However, despite the consistent progress in the definition of the molecular basis of ESCC, precision oncology for these patients is still virtually absent. The recent identification of molecular subtypes of ESCC with clinical relevance may foster the development of new therapeutic strategies. It is estimated that about 40% of the genetic alterations observed in ESCC are actionable. Furthermore, the recent introduction of solid tumor immunotherapy with immune checkpoint inhibitors (ICIs) showed that a minority of ESCC patients are responsive, and the administration of ICIs, in combination with standard chemotherapy, significantly improves overall survival over chemotherapy in ESCC patients with advanced disease.

## 1. Introduction

Esophageal cancer (EC) is the seventh most common malignancy worldwide, with more than 500,000 new cases diagnosed each year [1]. It is the sixth most frequent leading cause of cancer-related death each year [1]. The survival rate of EC patients is limited, with less than 20% of patients surviving 5 years [2,3].

Two main histological types of EC have been observed, esophageal adenocarcinoma (EAC) and esophageal squamous cell carcinoma (ESCC), exhibiting different molecular abnormalities, different risk factors and considerable geographic variation [2,3,4].

ESCC is the most frequent EC in the world, with the highest incidence in eastern Asia and parts of Africa; EAC is the most frequent subtype in Western countries [2,3].

Epidemiological studies have identified some lifestyle and environmental factors associated with high ESCC incidence: tobacco smoking and alcohol consumption, two factors that act synergistically in increasing the risk of developing ESCC; the consumption of hot beverages, poor diet and opium smoking are other factors that increase the risk of ESCC development; and other risk factors are polycyclic aromatic hydrocarbons, present in air or in contaminated foods or drinks [5,6,7].

Studies carried out over the last ten years have consistently contributed to a better understanding of the cellular and molecular mechanisms underlying both EAC and ESCC. Particularly, developments in high-throughput genomic technologies have allowed for a detailed characterization of the genetic abnormalities that occur in ECs, showing their genetic complexity and heterogeneity [4].

The present review will analyze the recent developments in the characterization of the molecular defects of ESCCs, attempting to define their complexity and heterogeneity; with the identification of some subgroups, they could be amenable to molecularly targeted treatments. Furthermore, the introduction of immunotherapy with immune checkpoint inhibitors (ICIs), in association with chemotherapy, seems to offer consistent chances for improved therapeutic responses in ESCC patients with advanced disease.

## 2. Precursor Lesions of ESCC

Studies on the early stages of ESCC showed that ESCC development is preceded by early changes of esophageal epithelium consisting of initial squamous hyperplasia (ESH), followed by squamous dysplasia (mild-, moderate- and severe-grade), which then develops into invasive cancer. Epithelial squamous dysplasia (ESD) is the only histopathological lesion that predicts the development of ESCC. ESD is found in 25% or more of adults above the age of 35 years in populations living in northcentral China, where the risk for ESCC is among the highest in the world [8].

In some areas of China, the risk of developing ESCC is high, and, therefore, programs for ESCC screening have been developed. In this context, a recent study reported the results of endoscopic screening carried out on 55,727 adults in the rural Chinese region of Feicheng: 3731 subjects had esophageal lesions classified as ESD, and 1753 of these subjects were followed for the time to monitor their ESD lesions [9]. A median follow-up of 2.3 years showed that 62.2% of these patients had a regression in their ESD lesions, 29.7% remained stable and 8.2% progressed. The median time for progression to ESCC for patients with mild ESD was 3.5 years; for those with mild ESD, it was 2.3 years, and for those with severe ESD, it was 2.2 years [9]. The probability per year of patients with mild ESD progressing to moderate ESD was 0.025, and the chance of regression to normality was 0.061; for those with moderate ESD, the chance of progression to severe ESD was 0.038, and the chance of regression to mild ESD was 0.194; finally, for those with severe ESD, the probability of progression to ESCC was 0.016 and of regression to moderate ESD was 0.11 [9].

Liu et al. reported an evaluation of genetic alterations in 227 tissue specimens derived from 70 patients undergoing tumor resection and analyzed normal tissues and tissues with simple hyperplasia, squamous dysplasia and ESCC tissues [10]. The comparative analysis of these tissues showed a mutation rate in exon regions corresponding to 4.55, 4.56 and 0.81 mutations/Mb for ESCC, ESD and ESH, respectively; the copy number alterations (CNAs) were 758, 420 and 29/Mb, respectively [10]. An analysis of polyploidy showed that 68% of ESCC, 55% of ESD and 0% of ESH were polyploid [10]. The mutation signatures were similar in ESD and ESCC. Large-scale chromosome deletions at 9p21.3 (*CDKN2A*) and 2q35 (*ASCL3, FEV*) and amplifications at 11q13.3 (*CCND1*), 5p15.33, 8q24 and 2q31.2 (*NFE2L2*) were common in both ESD and ESCC; however, ESCC contained more recurrent copy number alterations that ESDs [7]. The spectrum of mutated genes and their mutation frequencies was similar in both ESDs and ESCCs, and apparently, there are no gene mutations specific to ESCC compared to ESD [10]. In esophageal hyperplastic lesions, there is a much lower number of gene mutations (limited in a few cases to *TP53, CDKN2A* and *NOTCH1*), and no copy number alterations were observed. The shared genetic alterations observed in matched ESD and ESCC samples were considered trunk events and allowed for the identification of driver genes underlying the development of ESCC from ESD; three categories of trunk genes were identified, including DNA repair and apoptosis genes (such as *TP53* and *CDKN2A*); proliferation genes and oncogenes; cell adhesion; and junction genes [10].

Chen et al. reported the analysis of the genomic alterations of 45 ESDs synchronously observed with concomitant ESCCs and 13 ESD samples observed in the absence of ESCC [11]. The tumor mutation density (TMD) was comparable in ESCC (3.9 mutations/Mb) and high-degree ESD (4.1 mutations/Mb) and lower in low-grade ESD (3.3 mutations/Mb); importantly, TMD was lower in ESD lesions without ESCC compared with those associated with ESCC [11]. At the mutational level, both ESCC lesions and ESCC similarly displayed a strong enrichment of C > T transitions, mostly corresponding to mutational signature 1 [11]. The paired ESD and ESCC exhibited a highly comparable mutational landscape; ESD not associated with ESCC displayed a lower mutational profile (*TP53* mutations in 30.8% compared with 95.6% and 97.8% in tumor-associated ESD and ESS, respectively) [11]. Patients displaying multiple ESD lesions in association with ESCC allowed for the classification of gene mutations as trunk mutations (observed in all samples from individual patients), shared mutations (detected in more than one but not in all samples) and private mutations (observed only in one sample); all patients displayed branched clonal evolution [11]. Copy number alterations were observed both in tumor-independent and tumor-associated ESDs, but their numbers were lower in the former ones compared with the latter ones [11]. The analysis of the distribution of genetic alterations in tumor-independent, tumor-associated ESD and ESCC allowed for the proposal of a two-hit event to explain ESCC development based on precursor lesions: the loss of heterozygosity or mutations in *TP53* occurs early (being detectable in a part of tumor-independent ESD), but the subsequent development of ESCC requires the full inactivation of *TP53* [11]. This model explains why only a small proportion of ESDs finally develop into ESCC [11].

In 11 patients, Liang et al. evaluated the genome profiles using whole-exome sequencing in physiologically normal mucosa (PNM), ESCC cancer tissue and PMN samples from non-ESCC gastric cancer [12]. Significant differences in the mutation frequencies of *NOTCH1* and *NOTCH2* (more frequent in PNM than in ESCC); copy number variations at both the gene and chromosomal arm level; and mutations in cancer-related HIPPO, WNT and NRF2 signaling pathways (more frequent in ESCC than in PNM) were observed. Importantly, the frequencies of *TP53* gene mutations were higher in PNM derived from ESCC cases compared with those observed in PNM derived from gastric cancer patients [12].

Since most patients with ESD do not progress to ESCC, it is important to identify biomarkers predicting tumor progression in these individuals. In this context, a recent study compared tumor genomic alterations in two groups of ESD patients, one progressing to ESCC and the other one not progressing to ESCC: progressors had more somatic mutations and CNA burden, as well as APOBEC and age-related signatures at higher levels compared with non-progressors. Furthermore, a gene score consisting of a *NOTCH1* mutation and *CDKN2A* deletion status (the absence of a *NOTCH1* mutation and *CDKN2A* deletion) was predictive of progression in esophageal lesions, which show the absence of iodine-staining under endoscopy but have no or mild dysplasia [13].

Importantly, a study of the esophagus epithelium of normal individuals showed that somatic mutations accumulate with age in the absence of any evidence of tumor formation [14,15]. Thus, Martincorena et al. performed an ultra-deep targeted sequencing of 844 samples of normal esophageal epitheliums obtained from 9 deceased organ transplant donors, ranging from 20 to 75 years of age [14]. The sequencing of these samples showed the frequent occurrence of mutations, whose number per sample and allelic frequencies consistently changed across individuals, with the number of mutations and the sizes of mutant clones increasing with donor age [14]. The most frequently mutated genes were *NOTCH1, TP53, NOTCH2, FAT1, ARID1A, KMT2D, CUL3, AJUBA, PIK3CA, ARID2, TP63, NFE2L2* and *CCND1*; these genes showed consistent evidence of positive selection [14]. Eleven of these fourteen genes, found under positive selection in normal esophagi, are canonical drivers of ESCC. One of the unexpected findings of this study consisted of frequent *NOTCH1* mutations (enriched in truncating mutations) found in normal esophageal epitheliums, corresponding to about 120 different *NOTCH1* mutations per cm^2^ of a normal esophagus. The number of *NOTCH1* mutations increased with age, as supported by the observation that 30% to 80% of normal esophagi had *NOTCH1* mutants in five out of six middle-aged or elderly subjects, compared with 1% to 6% in three individuals under 40 years of age [14]. *TP53* is the second most frequently mutated gene in normal esophagi, with about 35 mutations per square centimeter and strong positive selection for both truncating and missense *TP53* mutations [14]. To evaluate the extent of selection driving clonal expansions operating in normal esophagi, the ratio of non-synonymous to synonymous (dN/dS) mutation rates for the different genes found to be mutated in normal esophageal epitheliums was evaluated. This analysis showed clear evidence of selection during clonal expansion in normal esophagi for the 74 genes investigated: dN/dS ratios for missense and protein-truncating mutations were 2.2 and 8.6, respectively, with the enrichment of non-synonymous mutations increasing rapidly with clone size [14]. The number of mutations found in normal esophageal epitheliums was related to the age of individuals and to smoking status [14]. According to the findings of this study, the high frequency of *NOTCH1* mutations in normal esophageal epitheliums, compared with the low frequency of *NOTCH1* mutations in ESCC, suggests that these cancers predominantly originate from the epithelium without *NOTCH1* mutations; in contrast, *TP53* mutations observed at a lower frequency than *NOTCH1* mutations in normal esophageal epitheliums, but present in the vast majority of ESCCs, suggest that these tumors originate from epithelial cells bearing these mutations [14].

In a subsequent study, the same authors explored the mechanisms underlying mutational selection using the study of the esophageal epitheliums of mutagen-treated mice as a model (the administration of diethylnitrosamine (DEN), a mutagen present in tobacco smoke, induces several mutations in the mouse esophageal epithelium); in these mice, deep sequencing identified a consistent number of mutant clones with multiple genes under positive selection, including *NOTCH1, NOTCH2* and *TP53* [14]. Transgene lineage tracing studies showed a marked clonal competition evolving in the time dictated following a model of spatial competition, implying that the proliferative advantage of positively selected mutations is dependent on neighboring cells. When the fitness of different clones is similar and these clones collide, the mutant cell rate reverts toward normal tissue homeostasis, and this condition explains how selection operates in normal esophageal epitheliums [15]. Although most esophageal epitheliums remain normal after DEN administration, the formation of discrete premalignant tumors scattered across a normal esophageal epithelium can be induced with time [15]. The rate of evolution of these premalignant tumors was then explored, showing that their evolution and survival depend not only on the mutations that they carry but also on the mutational landscape of the neighboring normal tissue: the majority of these newly formed esophageal tumors are eliminated through competition with mutant clones in the adjacent normal epithelium [16]. Thus, the number of tumor foci decreases from 588 10 days after treatment with DEN to 42 18 months after treatment with DEN, but the size of the surviving tumors increases from 5000 to 200,000 µm^2^; thus, >90% of the tumor foci are eliminated, and a marked development occurs in the surviving tumors [16]. Importantly, early tumors are basically polyclonal, while late tumors are essentially monoclonal; furthermore, early tumors display *NOTCH1* and *ATPA2* mutations with a frequency of 100% and 5%, respectively, while late tumors exhibit a frequency of 6% and 50% for the mutations of these two genes [16]. These findings show evidence of selection in surviving tumors [16]. The competition hypothesis is experimentally supported by the observation that the induction of highly competitive clones in transgenic mice increased early tumor removal, while the pharmacological inhibition of clonal competition reduced tumor removal [16].

A parallel study by Yokoyama et al. [17] confirmed the findings observed by Martincorena et al. [15]. Basically, they observed the progressive age-related expansion of the clones of epithelial esophageal cells, which carry mutations in driver genes such as *NOTCH1*, a phenomenon significantly accelerated by smoking and alcohol consumption [17]. Driver-mutated clones emerge multifocally, starting from early childhood and markedly increasing in number and size with aging, almost completely replacing the normal epithelium in very old individuals [15]. Some of the mutations observed in normal esophageal epithelium occur at a frequency higher than that observed in ESCC; this condition is particularly evident for *NOTCH1* mutations, but also for *PPM1D* (observed in 12.7% of normal epithelium compared with 0.58% in ESCC) and, to a lesser extent, for other genes, including *NOTCH2, ZFP36L2, FAT1, NOTCH3, CHEK2* and *PAX9* [17]. Lifestyle affects the frequency of some genes in normal esophageal epithelium, a phenomenon particularly evident for *PPM1D, EP300, TP53* and *NOTCH2* [17]. Copy number alterations are less frequent in normal esophageal epithelium than in ESCC, but their frequency markedly increases with aging; these copy number alterations occur at the level of a small number of chromosomes [17]. The most frequent CNA is represented by the uniparental disomy (UPD) of chromosome 9q, affecting the *NOTCH1* locus: this UPD causes LOH of *NOTCH1* and is invariably associated with *NOTCH1* mutations [17].

Three recent studies have contributed to a better definition of the role of *NOTCH1* and *TP53* mutations in normal esophageal epitheliums and in the generation of ESCC. Thus, Abby et al. evaluated the hypothesis, suggesting that *NOTCH1* mutations may promote clonal expansion but impede esophageal carcinogenesis [18]. First, they showed that *NOTCH1* mutant clones in aging esophagi display frequent biallelic mutations blocking NOTCH signaling; in mouse esophagi, heterozygous *NOTCH1* mutations confer a competitive advantage over WT cells, and this effect is enhanced by the loss of the second *NOTCH1* allele [18]. In carcinogenesis models, *NOTCH1* mutations are less frequent in tumors than in normal esophageal epitheliums; furthermore, *NOTCH1* deletion reduces tumor growth, as well as the pharmacologic inhibitors of NOTCH1 [18]. According to these findings, it can be concluded that *NOTCH1* mutations in normal esophageal epitheliums are beneficial [18].

The studies carried out on normal esophageal cells have shown that heterozygous *TP53* mutants are observed in normal esophageal epitheliums, reaching a frequency of 5–10% of tissue by middle age and then rising to 15–30% in elderly individuals, a finding suggesting that *TP53* mutations confer a competitive advantage over the esophageal cells not carrying these mutations [19]. In elderly individuals, rare clones bearing both a *TP53* mutation on one allele and a *TP53* LOH on the other allele have been observed [15]. Despite the accumulation of *TP53* mutations, the esophageal epithelium maintains a normal morphology and differentiation. The majority of ESCC samples carry *TP53* mutations on one allele concomitantly with a loss of heterozygosity on the other allele, thus resulting in biallelic *TP53* inactivation, a genomic alteration permissive for consistent genomic instability that allows the survival of tumor cells with large genomic alterations. In transgenic mice expressing mutant *TP53* at the level of esophageal progenitor cells, a proliferative advantage is observed with the expansion of mutant clones [19]. During the carcinogenic process, the *TP53* mutant does not initiate tumor formation, but tumors developing from areas exhibiting biallelic *TP53* inactivation develop larger tumors in a display of chromosomal instability, which are conditions that favor ESCC development [19]. According to these findings, a model of ESCC development was proposed implying that heterozygous *TP53* mutations at the level of single cells in normal esophageal epitheliums confer a proliferative advantage, allowing for clonal expansion, and a population of these cells persisting over time may undergo LOH at the level of a normal *TP53* allele; most cells with LOH are lost due to neutral competition, but few mutant clones persist over time and develop genome instability, acquiring CNAs, and may progress to form ESCC [19].

Fernandez-Antoran et al. showed that environmental conditions may exert selective pressure on *TP53*-mutant esophageal cells [20]. Particularly, they explored the effect of oxidative stress induced by low-dose ionizing radiation (LDIR) on WT and *TP53*-mutated cells in transgenic mouse esophagi, showing that this treatment stops the proliferation and differentiation of WT cells, while *TP53*-mutant cells are insensitive to this treatment and outcompete normal esophageal cells [20]. Importantly, the association with the antioxidant treatment, together with LDIR, reverses this effect, promoting the proliferation of WT cells and reducing *TP53*-mutant cells [20].

## 3. Genetic Alterations of ESCC

### 3.1. Genomic Alterations of ESCC

Studies carried out over the last ten years have provided a detailed characterization of molecular alterations occurring in ESCCs, showing their complexity and heterogeneity. ECs are among the human solid tumors with the highest number of molecular alterations [21].

Numerous studies, mainly based on next-generation sequencing, have provided a characterization of the molecular alterations occurring in ESCCs and involving gene mutations, gene amplifications, gene deletions and chromosome rearrangements [22,23,24,25,26,27,28,29]. The most frequent mutations occur at the level of the *TP53, NFE2L2, NOTCH1, PIK3CA, ZNF50, NOTCH3, TGFBR2, KMT2D, TRRAP, PTCH1, SMARCA4, LRP1B, HDAC4, KMT2C* and *PCLO* genes; the most frequent copy number alterations (amplification or deletion) occur at the level of *CCND1, CDKN2A, CDKN2B, FGF3, FGF4, FGF19, PIK3CA, TBL1XR1, TERC, MECOM, LPP, MTAP, PRKCL* and *EGFR* genes [22,23,24,25,26,27,28,29]. It is of interest to note that, although ESCC and EAC display some similarities in their molecular alterations, they have many marked differences, supporting the view that they are two different diseases.

The comparative analysis of the genomic abnormalities of ESCC and EAC shows that these two tumors display few genetic abnormalities with similar frequencies, such as *TP53* and *KMT2C* mutations, while the vast majority display consistent differences: *KMT2D, NFE2L2, NOTCH1* and *PIK3CA* gene mutations are more frequent in ESCC than in EAC; *CDKN2A* and *ARID1A* mutations are less frequent in ESCC than in EAC; *CDKN2A, CDKN2B, CCND1, FGF3, FGF4, FGF19, PIK3CA* and *EGFR* copy number alterations are more frequent in ESCC than in EAC; and *HER2, VEGFA* and *KRAS* copy number alterations are less frequent in ESCC than in EAC [30].

Two recent studies reported an integrative genomic analysis of a large cohort of published ESCC patients [31,32]. Zou et al. reported the integrated reanalysis of the mutation data of WGS or WES from a total of 1145 tumor samples present in 7 large ESCC cohorts [31]. In 1145 ESCC patients, an average of 4.3 mutations/megabase was observed. Using a cutoff of eight mutations/megabase to distinguish low tumor mutation burden (TMB) from high, it was shown that patients with high TMB have a lower OS compared with those with low TMB [31]. In the 7 cohorts of ESCC patients included in this analysis, 47 mutated genes were considered driver genes, but only 7 genes (*TP53, NOTCH1, CDKN2A, ZNF50, NFE2L2, PIK3CA* and *RB1*) were considered driver genes in the majority of studies; some genes, such as *FAT1, FAT2, PTEN, EP300, FBXW7, APS31, MUC16* and *RP&15*, were considered driver genes only in a minority of studies [31]. Only the mutations of a few genes were correlated to clinical parameters, such as *ZNF50* mutations related to lymph node metastasis [31].

Li et al. reported a large meta-analysis of molecular data relative to 1930 ESCC genomes from 33 datasets [32]. The most frequently mutated genes were *TP53* (78%), *TTN* (35%), *MUC16* (16%), *NOTCH1* (16%), *COSMO3* (15%), *KMT2D* (12%), *FAT1* (10%) and *LPR1B* (10%) [32]. The median number of non-synonymous mutations was 81, which increased with the tumor stage. The classification of mutations according to their functional category showed that 38% of the patients displayed at least one mutation in the Hippo pathway (*FAT1, FAT2, FAT3*), 38% with histone modification (*KMT2D, EP300, KMT2C*), 33% in the NOTCH pathway (*NOTCH1, NOTCH3, FBXW7*), 27% in the DNA repair pathway (*BRCA2, TDG, FANCM, RIF, ATM*), 19% in the RTK-RAS pathway (*ERBB4, ROS1, RASA1, ALK*), 17% in the cell cycle (*CDKN2A, RB1*), 15% in the PI3K pathway (*PIK3CA*) and 12% in the NRF2 pathway (*NFE2L2, KEAP1*) [30]. Importantly, 14.7% of the patients display mutations in at least one druggable gene, including *BRCA2* (3%), *BRCA1* (2%), *ROS1* (2%), *ALK* (2%), *EGFR* (3%) and *HER2* (1%). The mutation of some genes was shown to be associated with clinical characteristics: the mutational frequencies of *NOTCH1, NOTCH3* and *XIRP2* were significantly higher in old patients, while the young patients displayed more frequent *RB1* and *PKHD1L1* mutations; the ESCC tumors localized in the upper thoracic part possessed a higher mutational load compared with those localized in the middle or lower thoracic part; *CDKN2A, LAMA3* and *NALNCN* mutations were associated with a better prognosis in early-stage patients, while mutations in *NFE2L2, FBN2, RNF213* and *ATP10D* were associated with a poor prognosis in late-stage patients [32]. The combination of the eight genes (*NFE2L2, CSMD1, CREBBP, KALRN, PRUNE2, NRXN1, AKAP9, FREM2*) with more pronounced prognostic predictivity in a unique panel allowed the opportunity to define a mutational score and to evaluate its prognostic impact on the whole population of ESCC patients showing that patients with one mutation in one of these eight genes have an intermediate prognosis, while those with two or more mutations have a lower survival compared to those with no mutations in these eight genes [32].

### 3.2. Mutational Signatures in ESCC

Somatic mutations in cancer genomes are caused by different and multiple mutational processes, each of which generates a characteristic mutational signature; the various tumors differ in their mutational signatures [33]. Mutational signatures are related to environmental or endogenous mechanisms [34].

Several studies have characterized the most recurrent mutational signatures observed in ESCC. In a TCGA characterization of ECs, it evidence showed that ESCC displays the enrichment of C > A substitutions and APOBEC (apolipoprotein B mRNA editing enzymes, catalytic polypeptide-like) signatures [27]. In their analysis of 508 Chinese ESCC patients, Cui et al. reported the occurrence of 11 mutational signatures: S1 and S2 were related to APOBEC; S3 to mismatch repair deficiency; S4 to age; S8 to aristocholic acid; S9 to alcoholic consumption; S11 to homologous recombination deficiency; and S6 was similar to the COSMIC 17 signature and was found in association with gastric acid reflux. S1, S2 and S11 were more common among patients with advanced disease; S3, S4, S9 and S10 were more frequent among patients at earlier stages [35]. Clustering analyses of these molecular signatures, according to nonnegative matrix factorization, allowed for the identification of three clusters: cluster 1 was characterized by APOBEC signatures (S1 and S2); cluster 2 shared features with deficient homologous recombination repair signatures (S6 and S11); and cluster 3 was characterized by mismatch repair deficiency (S3) and the spontaneous deamination of 56-methylcytosine (S4) [35]. Patients pertaining to cluster 1 have a shorter overall survival rate compared to those in clusters 2 and 3 [35]. *ZNF50* mutations are highly enriched in cluster 1 [35].

Li et al., in their wide analysis of 1930 ESCC samples, analyzed mutational signatures via NMF and defined 11 mutational signatures, with 5 signatures (Sig 1, Sig 2, Sig 4, Sig 6 and Sig 8) dominating > 90% all patients [32]. Sig 1 is characterized by C > T mutations, and it includes many patients with a smoking and drinking history and is associated with a significantly worse prognosis compared with other signatures. Sig 2 is a major contributor (44.7% of all cases), is caused by the spontaneous deamination of 5-methylcytosine and is associated with age. Sig 7 and Sig 8 are related to the activity of APOBEC and contribute to 16.8% of all cases; these signatures correlate with the mutational load. Sig 4 is characterized by the failure of DNA double-strand break repair, displays a negative correlation with the mutational load and is not related to BRCA 1–2 mutations [32].

Li et al. performed an analysis of mutational signatures in 549 ESCC samples and observed the prevalence of 3 signatures: C > T at CpG, C > T at TpCp[A/T] (APOBEC) and T > C; the signature, characterized by T > C mutations, is significantly associated with alcohol consumption [29]. Another study on the whole-genome sequencing of DNA and RNA in 94 Chinese patients with ESCC identified 6 mutational signatures (E1 to E6) [31]. One of these signatures, signature 4, is unique in ESCCs linked to alcohol intake and genetic variants in alcohol-metabolizing enzymes; this signature, specifically characterized by T > C mutations (seemingly caused by the differences in repair efficiency of DNA damage and maintained by processes between the transcribed and untranscribed strands of genes), is like COSMIC Signature 16 and is significantly associated with an individual’s smoking and drinking status [31]. Interestingly, the frequency of signature E4 mutations in the ESCCs of drinkers with the risk *ALDH2* genotype (rs671-AG/-AA) was significantly higher than that of drinkers with the non-risk genotype (rs671-GG) or non-drinkers with or without the risk *ALDH2* genotype [31].

### 3.3. Molecular Classification of ESCC

Many studies on the molecular characterization of ESCC have proposed different molecular characterizations of ESCCs based on genomic and transcriptomic criteria.

TCGA proposed a classification of ESCC subdivided into three subtypes called ESCC1, ESCC2 and ESCC3. ESCC1 is characterized by the recurrent alteration of the NRF2 pathway, regulating the adaptive response to oxidative stressors. Alterations have been observed in *NFE2L2* (*NRF2*, 30%), *KEAP1* (6%) and *CUL3* (10%) genes involved in NRF2 degradation and in the *ATG7* (12%) gene-encoding of an NRF2 pathway autophagy factor [27]. Genes involved in the control of cell differentiation, such as *SOX2* and *TP63* amplification, are more frequent than those observed in other ESCC subtypes. Finally, ESCC1 displays higher rates of *YAP1* amplification and *VGLL4/ATG7* deletion, suggesting the possible activation of the Hippo signaling pathway. The ESCC2 subtype is characterized by higher rates of *NOTCH1* or *ZNF50* mutations; more frequent inactivating alterations of *KDM6A* and *KDM2D*; and *CDK6* amplification, and inactivation of *PTEN* or *PIK3R1*. Alterations of the NRF2 gene pathway are rare in ESCC2, while alterations of genes involved in cell differentiation are frequent (50% of cases), with *SOX2* and *TP63* amplification occurring in 36% of cases [27]. The ESCC3 subgroup did not show evidence of the genetic deregulation of the cell cycle and displayed *TP53* mutations in only 25% of cases, while alterations of genes in the PI3K pathway are frequent, accounting for 75% of cases, including *KMT2D/MLL2* and *SMARCA4* gene alterations [27].

Du et al. reported the molecular characterization of 490 Chinese ESCC cases and, according to the profile of the copy number alterations, proposed a classification of these tumors into three subtypes; among these subtypes, ESCC subtype 3 displayed a more elevated number of CNAs than the other two subtypes [28]. Particularly, subtype 3 displayed more frequent *PIK3CA* amplification (71.8% vs. 15.7%) and *FBXW7* deletion (46.8% vs. 0%) than subtype 2 tumors [28]. Li et al., through the analysis of a quality-controlled integrated ESCC genomic dataset of ESCC-META cohort, evaluated the genomic features underlying clinical characteristics and identified 11 mutational signatures in ESCC, some of which were related to clinical features [32]. 

Liu et al., through an integrated genomic and transcriptomic analysis of 125 Chinese ESCC patients, proposed a molecular classification involving three subtypes, in part different with respect to those reported in the study by TCGA [36]. According to the mutational profile and, mostly, the gene expression profile, they distinguished three ESCC subtypes: ESCC subtype 1 more frequently displays *GPR98, DDX60* and *DDX60L* mutations, and at the transcriptional level, it is characterized by the highest level of metabolism-related pathways and the drug metabolism cytochrome p450 signaling pathway; ESCC subtype 2 is more likely to have *NPIPA* and *MGAM* mutations, and at the transcriptional level, it is characterized by the expression of the epithelial-to-mesenchymal transition pathway and the expression of immune inhibitors; ESCC subtype 3 is characterized at the genomic level by more frequent *NOTCH1, KMT2D* and *MIB2* mutations, and at the transcriptional level, it is characterized by an increased expression of cell-cycle-related pathways and WNT activation [36]. This pattern of gene expression suggests the potential sensitivity of subtype 2 tumors to immunotherapy with immune checkpoint inhibitors and subtype 3 tumors to cyclin inhibitors, such as CDK4-CDK6 inhibitors [36].

Liu et al. proposed classifying ESCC into two subtypes, S1 and S2, based on an analysis of proteomic and phosphoproteomic profiles [37]. The two subtypes differ in the degree of upregulation or downregulation in a set of proteins that is more pronounced in S2 than in the S1 subtype: the most upregulated proteins in S2 are enriched in pathways such as mRNA processing, DNA replication, DNA repair, E2F targets and the G2/M checkpoint; the most downregulated proteins involve pathways such as extracellular structure organization, extracellular matrix organization and focal adhesion. Patients with the S2 subtype have worse overall survival and disease-free survival outcomes compared with those with the S1 subtype [37].

Cui et al. have explored the genomic abnormalities of 508 ESCC patients and proposed a classifying these tumors into three subgroups: NFE2L2-mutated, with poor prognosis; RTK-RAS-MYC-amplified (tumors with receptor tyrosine kinase/RAS amplification, such as EGFR or FGFR1 amplification, comprising about 50% of cases), with intermediate prognosis; and double-negative, with a better prognosis [35].

Recently, Mai et al. reported the results of a targeted deep sequencing study to discover ESCC subtypes, and using this approach, they identified a poor prognosis subgroup of patients characterized by frequent mutations related to the Hippo pathway [38]. According to the distribution of prognosis-associated mutations, three clusters of patients were identified using a clusterization algorithm: WSCC1, WSCC2 and ESCC3 [38]. ESCC1 is characterized by recurrent mutations in *FLG* (62%), *AHNAK2* (45%) and *EP300* (34%); EP300 is a putative driver of the NOTCH pathway. ESCC2 is characterized by recurrent mutations in *USH2A* (46%) and *AHNAK* (44%). ESCC3 is characterized by frequent *FAT1* (55%), *FAT3* (25%) and *FRY3* (37%) mutations; these three genes are major regulators of the Hippo pathway [38]. Particularly, *FAT1* is a tumor-suppressor that dampens the activity of the YAP Hippo effector; *FAT3* is a homologous protein to *FAT1*; *FRY* is another inhibitor of YAP, blocking its nuclear translocation. Based on these findings, a three-gene mutation signature was developed: patients with one or more mutations in these three genes were assigned to the FAT/FRY subgroup, and it was shown that they have a poor survival rate compared with patients with WT cells for these mutations (mOS 22.8 months and vas 39.6 months, respectively) [37]. Furthermore, patients with co-mutated *FAT3* and *FRY* genes have a particularly poor prognosis [38]. At the molecular level, the FAT/FRY subgroup does not show enrichment in NOTCH or NRF2 pathway mutations and shows the decreased expression of Hippo pathway-related genes [37]. Since the tumors of the FAT/FRY subgroup display increased CD8^+^ T cell infiltration and activated dendritic cells, as well as high tumor mutation and neoantigen burdens, it has been suggested that they could be sensitive to immunotherapy treatments [38].

### 3.4. ESCC Genetic Alterations and Response to Chemoradiotherapy

The standard treatment of ESCC patients with locally advanced disease involves, in addition to surgery, treatment with radiotherapy combined with chemotherapy (5-fluorouracil plus cisplatin); only about 30% of patients survive for 5 years after this treatment.

Few studies have explored the genetic alterations associated with the response or resistance to chemoradiotherapy. Hirata et al. reported the study of 52 tumor samples from 33 patients, with ESCC receiving combined treatments with radiotherapy and 5-fluorauracil/platinum [39]. An analysis of five patients at the level of pretreatment and locally recurrent lesions showed that most driver-gene-altered clones survived chemoradiotherapy treatment and only a few driver-gene alterations were acquired during recurrences [39]. The mutational signatures markedly changed after treatment, for example, with an increased frequency of deletions and platinum dose-dependent base substitution signatures [39]. A multiregion analysis of the ESCC tumors of 28 patients showed that copy number gains of the *MYC* locus, responsible for *c-myc* amplification, are associated with poor PFS and OS; *c-myc* amplification remained during the course and was potentially identified as a main genetic mechanism of resistance to chemoradiotherapy [39]. In line with this hypothesis, *c-myc* amplification and protein levels in the ESCC cell line improved their radiosensitivity [39]. Other studies support the prognostic significance of *c-MYC* amplification in ESCC. Thus, Huang et al. reported the occurrence of *c-MYC* gene amplification in 43% of patients with ESCC; in patients with *c-MYC* amplification, the PFS and OS medians were 24 and 31 months, compared with 48 and 48 months, respectively, for patients without *c-MYC* amplification [40]. Furthermore, in these patients, *c-MYC* amplification correlated with age and lymph node metastases [40].

Weng et al. reported the results of a whole-exome sequencing analysis on paired tumors obtained before and after radiotherapy from 11 ESCC patients [41]. This comprehensive analysis showed that the tumor mutation burden was similar before radiotherapy (12.5 mutations/Mb) and after radiotherapy (13.5 mutations/Mb); no differences were found in the number of single nucleotide variants in the ploidy average or in the frequency of amplifications, deletions and LOH regions between pre-radiotherapy and post-radiotherapy patients [41]. The frequency of mutations in the most recurrent driver cancer genes remained unchanged after radiotherapy except for the *EPHA2* (Ephrin-A2) gene, whose mutation frequency was significantly reduced after radiotherapy [41].

### 3.5. Intratumoral Heterogeneity of ESCC

In addition to intertumor variation, ESCCs also display a consistent degree of intratumor heterogeneity, as assessed through multiregion sequencing studies of tumors. Intratumor heterogeneity is a complex phenomenon dependent on the presence of different molecular clones within a single tumor.

Hao et al. reported the results of a fundamental study involving multiregion whole-exome sequencing and global methylation profiling in thirteen patients with primary ESCC; for each tumor, four different regions were analyzed [42]. Phylogenetic trees were constructed based on mutations—including both non-silent and silent mutations—identified in each tumor. In total, 35.8% of somatic mutations and 90% of recurrent copy number alterations were found to be spatially heterogeneous [42]. Driver mutations were more enriched in trunks compared with passenger mutations; the subdivision of driver mutations in mutations occurring in oncogenes (such as *PIK3CA, KIT, NFE2L2* and *MTOR*) and mutations occurring in tumor suppressors (such as *TP53*) showed that the former ones are preferentially enriched in branches, while the latter ones are truncal [42]. An evaluation of the clonal status of somatic mutations showed that cancer-related genes found on the trunks, such as *TP53, NOTCH1, KMT2D,* and *CREBBP,* are predominantly mutated in a clonal manner; in contrast, other driver mutations, such as those occurring in *MTOR, KEAP1, PTPRB* and *FAM135B*, are subclonal. Interestingly, in some patients, *PIK3CA, KIT* and *NFE2L2* mutations are clonal in some tumor areas but absent in other ones, thus indicating a mixed and complex intratumoral clonal status. At the level of mutations that can be therapeutically targeted, mutations at the level of *KIT, AURKA* and *CCND2* are always branched/subclonal, while *ERBB4, FGFR2, BRCA2, ATM* and *TP53* are truncal/clonal [42]. A study of intratumor heterogeneity at the copy number level showed the existence of an extensive heterogeneity for CNA, with 90% of all recurrent CNAs being spatially heterogeneous [41]. The only driver CNA that was consistently ubiquitous was a copy number gain found at 11q13, encompassing a number of oncogenes such as *CCND1, ANO1* and *CTNN*, thus supporting the key role of this chromosome aberration in ESCC development [42]. A study of DNA methylation profiles at the tumor regional level showed a consistent level of intratumoral heterogeneity with the presence of multiple, epigenetically distinct, subclonal cell populations and a possible relationship between genomic and epigenomic alterations [42]. Thus, this study showed the consistent level of intratumoral heterogeneity occurring in ESCC, representing a key event in the mechanisms of the evolution of these tumors and consistently contributing to drug resistance and treatment failure.

Yan et al. reported the results of a multiregion sequencing study performed on 39 ESCC patients [43]. The mutational burden of the ESCC patients was higher in patients harboring larger tumors than those with smaller tumors; importantly, mutations per patient identified using multiregion sequencing were significantly higher than those observed using single-region mutational analysis [43]. According to their cellular distribution in tumor cells, the mutations were subdivided into trunk mutations (ubiquitous), shared (present in some but not all tumor regions) and private (present in only one tumor region). All 39 ESCC cases explored in this study displayed consistent spatial intratumor heterogeneity, with an average of 63% of somatic mutations being heterogeneous; tumor stage seemed to be negatively associated with the proportion of ubiquitous mutations, and significantly more heterogeneous mutations were observed in patients with metastatic lymph nodes [43]. Interestingly, the multiregion analysis showed that the mutational frequency of some mutated driver genes, such as *NOTCH1* (33%), *PIK3CA* (25%) and *KMT2D* (30%), was higher than that reported in a single-region mutational analysis. Some driver mutations, such as *TP53, PIK3CA, ZNF50* and *BRCA2*, have been found to be truncal mutations, while mutations in *NOTCH1, CREBBP, PTCH1, MET, MTOR* and *AXIN2* are usually found at the branches of clonal evolution trees [42]. Interestingly, 12.5% of tumors harbor *ERBB4* mutations, and functional studies support an oncogenic role for these mutations. A study of copy number alterations also showed a consistent degree of intratumor heterogeneity, with some CNA events being subclonal and emerging in the late stages of tumor development; genes involved in cell cycle regulation, such as *TP53*, *CCND1, RB1, CDK6* and *CDKN2A*, displayed early-occurring CNAs, while alterations of RTK/RAS/PI3K genes were usually late-occurring events [43]. Importantly, amplifications of the *EGFR* and *FGFR1* genes were frequently (50% of cases) heterogeneous in samples acquired from the same patient, and this finding had important implications since EGFR and FGFR1 are druggable genes [43]. In total, 18% of ESCC patients displayed early-occurring *BRCA1-2* mutations, associated with high levels of the mutational signature 3; knockdown of *BRCA1-2* strongly increased the sensitivity of ESCC cells to cisplatin. Interestingly, 5% of ESCC patients displayed a high level of CD274, which resulted in a high expression of PD-L1, a property that could render these patients more sensitive to therapy with immune checkpoint inhibitors [43]. Finally, a high correlation between genomic alterations and T cell receptor repertoire ITH (associated with a high level of neoantigens) was observed [43].

Most studies have considered gene mutations as binary variables, such as mutant versus wild type; however, a recent study showed that the cancer cell fraction (CCT) of mutations, a measure of intratumor heterogeneity, is more informative than the mutation status of genes in predicting disease recurrence after surgery in ESCC patients [44]. Mai et al. reported the results of a deep sequencing analysis of 201 ESCC patients aimed at determining the mutational status and CCF of mutations [44]. A binary analysis of the mutational status showed that 12 mutations and 19 gene-level CNAs were associated with disease recurrence [44]. An analysis of the prognostic impact of the CCF of mutated genes showed three different patterns: CCF-independent, CCF-dominant and CCF dose-dependent. For the CCF-independent pattern, the prognostic effects of the mutations were independent of the CCFs; for the CCF-dominant pattern, the prognostic effects of mutations can be observed when the CCFs exceed a threshold; for the CCF dose-dependent pattern, the more tumor cells that carry given mutations, the more notable the prognostic effect of the mutations [44]. Using sequencing data, a predictor model for disease recurrence after surgery was constructed, including eight genes, *GPR98, LAMA1, IFT140, MUC17, PTPRB, AHNAK2, PREX2* and *STAPA31D* [44]. The analysis of ESCC patients using this CCF-based predictor allowed for the identification of three risk groups of patients: high-, intermediate- and low-risk. The three-year disease-free survival rates were 6.3%, 29.8% and 70.5% in high-, intermediate- and low-risk patients, respectively [44].

### 3.6. Gene Expression Studies

Several gene expression studies have attempted to provide a better molecular classification of ESCC patients and identify new biomarkers to predict responses to current treatments and provide new prognostic criteria.

A pivotal study by Su et al. reported the first systematic analysis of gene expression profiling in a population of 53 ESCC patients and showed a consistent dysregulation compared with normal esophageal tissue, with 118 upregulated and 43 downregulated genes; immunohistochemical studies showed a pattern of protein expression concordant with mRNA quantification studies [45]. The upregulated genes involve multiple functional pathways, including extracellular matrix (with some mRNAs being markedly upregulated, such as MMP1, CTHRC1, SPP1, and COL1A2), cell adhesion, DNA replication, cell proliferation, cell cycle regulation and signal transduction; similarly, downregulated genes involve multiple functional pathways [45].

ESCCs, like other squamous tumors, are characterized by the increased expression of transcripts such as BNC1, DSC3 and DSG3 [46]. A hierarchical clustering of RNA profiling analysis allowed for the identification of three ESCC subtypes, which were distinguished by their cell cycle expressions and neural transcripts [46]. Subtype 1 can be further subdivided into two subgroups, 1a and 1b, which differ in the expression of transcripts associated with DNA replication and repair, small GTPases and homeobox genes [46]. These three subgroups, identified according to their RNA expression profiles, also differed in their genomic properties, in that subgroup 1b had the lowest somatic mutations per Mb (0.75) compared with subgroup 1a (11.8) and subtype 2 (3.71). Subgroup 1b also displayed a lower number of CNAs, with a decreased number of *MYC, EGFR* and *TP63* amplifications and *CDKN2A/B* deletions compared with the other subgroups; subgroup 1b was also characterized by a lower number of *TP53* mutations [46].

TCGA’s integrative analysis of ESCC proposed classifying these tumors into three molecular subtypes (ESCC1, ESCC2 and ESCC3); these tumors differ in their recurrent molecular abnormalities, but also in their gene expression and DNA methylation pathways [27].

Wang et al. identified two ESCC molecular subtypes in Asian populations using gene expression profiling analysis: subtype II ESCCs were enriched in pathways, including immune response, while genes overexpressed in subtype II ESCCs were enriched in pathways related to ectoderm development, the glycolysis process and cell proliferation [47]. Furthermore, some potential ESCC subtype-specific diagnostic markers were identified, including FOXA1 and ERYA2 for subtype I and LAMC2 and KRT14 for subtype II [47].

Zhang et al. reported the analysis of three publicly available gene expression profile datasets from the Gene Expression Omnibus (GEO) database; this analysis led to the identification of 345 differentially expressed genes, which were enriched in functional pathways related to the cell cycle, endocytosis, pancreatic secretion and fatty acid metabolism [48]. Twenty-one HUB genes with a significant influence on ESCC progression were identified, and five of these genes (*CPP1, BGN, SPARC, POSTN* and *Cd1A2*) were identified as having a high expression level associated with poor disease-free survival for ESCC patients [48]. In a subsequent study, the same authors analyzed 9n 9 GEO mRNA profiling datasets from ESCC patients and identified 152 differentially expressed genes involved in multiple processes in the microenvironment; this analysis showed that the M0 and M1 macrophages are markedly increased, while M2 macrophages are decreased [49]. Among these differentially expressed genes, nine Hub genes (*CDA, CXCL1, IGFBP3, MMP3, MMP11, PLAU, SERPINE1, SPP1* and *VCAN*) were identified; the expression of *MMP3* and *PLAU* correlated with macrophage infiltration. A seven-gene signature constructed from differentially expressed genes accurately predicted ESCC prognoses [49].

Several recent studies have explored the gene expression profile of ESCC in view of identifying some genes whose expression is prognostic. Thus, Li et al. explored the expression profiles of multiple ESCC datasets and, through the integration of these profiles, identified six upregulated genes (*HEATR1, TIMELESS, DTL, RUVBL1* and *ECT2*), which were found to be highly important in ESCC survival; the alterations in the expression of four genes (*PRIM2, HGPD, NELL2* and *RFAP28*) were associated with changes in chromatin accessibility [50]. The authors next explored intertumor heterogeneity and, using a regression and prognosis-related classifier, identified two tumor subtypes, S1 and S2, distinguished according to their outcomes and gene expression profiles. The S1 subtype comprises more aggressive tumors; exhibits pronounced stromal activation signatures with high scores in pathways such as TGF-beta signaling, vascular smooth muscle contraction, angiogenesis and fibroblast TGF-beta response signatures; and displays features of epithelial to mesenchymal transition. The S2 subtype comprises less aggressive tumors and activates fructose and mannose metabolism [50]. A random forest analysis showed that the *TNS1* gene was the top contributor to this survival classification of ESCC; *TNS1* was predominantly expressed in fibroblasts, and patients with a higher proportion of TNS1-positive fibroblasts in the tumor stroma have a poor prognosis and display lower CD8-positive T cell infiltration in the tumor parenchyma, associated with an immune exclusion phenotype [50]. Zhang et al. explored the expression of immune-related genes (IRGs) in ESCC and observed that, compared with normal esophageal tissue, ESCCs display 247 upregulated IRGs and 56 downregulated IRGs, and most differentially expressed genes are involved in cytokine-cytokine receptor interaction [51]. Nine of these genes (*HSPA6, SP100A12, CACYBP, NOS2, DKK1, DSM, STC2, NGPTL3* and *NRF2*) defined two ESCC subgroups: a high-risk subgroup with the enrichment of M0 and M2 macrophages and activated mast cells and a low-risk subgroup rich in CD8 T cells and regulatory T cells [51]. A recent study showed that the expression level of two genes may help define the prognosis of ESCC patients: ESCC patients with high *PDZK1P1* expression had worse prognoses than those with low expression; in contrast, *TM9SF1* expression levels were correlated with better prognoses [52]. The incorporation of the mRNA expression levels of these two genes with TMN staging and age allowed for the development of a prognostic model with good predictive accuracy for ESCC patients [52].

Other studies have evaluated the potential capacity of gene expression analysis to predict responses to neoadjuvant chemotherapy. Thus, Wen at al., through gene expression analysis, identified some genes whose expressions predict responses to neoadjuvant treatments with chemoradiotherapy (CCRT) in ESCC patients; six genes (*LIMCH1, SDPR, C1orf226, SLC9A9, GSMM3* and *IGSFIo*) were downregulated and four genes (*MMP1, MMP9, MMP12* and *OASL*) were upregulated in patients exhibiting a complete response compared with those achieving only a suboptimal response [52]. A simplified three-gene signature (*MMP1, LIMCH1* and *C1orf226*) was able to appropriately predict responses in 81% of tumor cases; particularly, all cases with suboptimal responses were identified, while only 54% of cases with complete responses were predicted [53].

A recent study explored the immunogenomic features of ESCC patients responding to platinum-based neoadjuvant chemotherapy (NAC); this study was performed on a cohort of 121 ESCC patients undergoing NAC treatment: 8 of these patients achieved complete responses and 67 partial responses and together were classified as responders, while 36 showed stable disease and 10 showed progressive disease, and they were considered no-responders [54]. A study of gene expression profiles using gene set enrichment analysis showed that the differential expression of immune-related genes is associated with responses to NAC; IL2-STAT5 signaling and interferon–gamma response are significantly enriched in responders [54]. The subdivision of T cell transcription signatures into hot, middle and cold showed that the patients with a hot signature comprised 88% of the responders, compared to 60% and 57% among the patients with middle and cold signatures, respectively [54]. The subclassification of the type of immune cells predominate in the tumor, as evaluated using gene expression profiling, showed that the CD8, CD4 and B cell classes have response rates to NAC of 73%, 74% and 75%, respectively, while the neutrophil class displayed a response rate of only 38%; however, compared with the neutrophil class, only the CD4 tumor class displayed a significantly prolonged overall survival rate [54]. Interestingly, in experimental models, neutrophil depletion promotes tumor sensitivity to NAC [54]. The tumor mutational burden was similar in responders and no-responders; the no-responders displayed enrichment in a mutational signature related to tobacco smoking [54]. The analysis of CNAs showed some remarkable differences between responders and no-responders, with rich chromosome 9p loss (with *CDKN2A*) deletion among no-responders [54]. The integration of smoking status, transcriptomic, CNV and immune characteristics allowed for the development of a machine learning model predicting the responses of ESCC patients to NAC [54].

The mechanisms that determine deregulated gene expression in ESCC are complex and not only related to genomic events but also to epigenetic changes. Concerning genomic events, alterations of histone-modifying genes (*KMT2D, KMT2C, EP300, CREBBP, SRCAP, KDM6A, KMT2A, NSD1, KMT2E, BAP1, ATRX, KDM3A*), found to be mutated in 38.5% of cases, are among the most frequently mutated genes in ESCC [33]. The Cancer Genome Atlas (TCGA) research group identified ESCC-related biomarkers using integrated genomic, epigenomic, transcriptomic and proteomic analyses and found evidence of 82 altered DNA methylation events, along with genomic and transcriptomic alterations [55].

A global methylome analysis provided evidence of pronounced alterations of DNA methylation, responsible for the deregulated expression of many genes [56]. Particularly, 98% of CpGs are hypomethylated in the ESCC genome; hypomethylated regions are genome areas with heterochromatin binding markers such as H3K9me3 and H3K27me3; hypermethylated regions are enriched in polycomb-repressive-complex-recognizing regions [56]. Alterations at the level of gene promoters, enhancers and gene bodies, as well as in polycomb repressive complex occupancy, are associated with the cancer-specific deregulation of gene expression [56].

## 4. Targeted Therapy in ESCC

### 4.1. Actionable Genetic Alterations in ESCC

An analysis of the genetic alterations observed in ESCC suggests that about 40% of these alterations are actionable. In particular, a recent study of deep target sequencing of 831 cancer-related genes in 118 ESCC patients showed that 43% of these patients had actionable genetic alterations: 17.8% of patients displayed the 11q13 amplicon (*CCND1, FGF3, FGF4* and *FGF19*), and these patients can be enrolled in clinical trials with FGFR inhibitor alone or in combination with CDK4/6 inhibitors; about 17% of patients displayed mutations in the NFE2L2/KEAP1/CUL3 pathway, eligible for clinical trials with glutaminase inhibitor; about 11.9% of patients displayed *PIK3CA* mutations, which may be targeted using specific inhibitors, such as alpelisib; and patients with *NF1, STK11* and *PTEN* mutations can be targeted with a MEK inhibitor or an mTOR inhibitor [57].

### 4.2. Therapeutic Targeting of RTK-RAS Pathway

**Figure 1:**
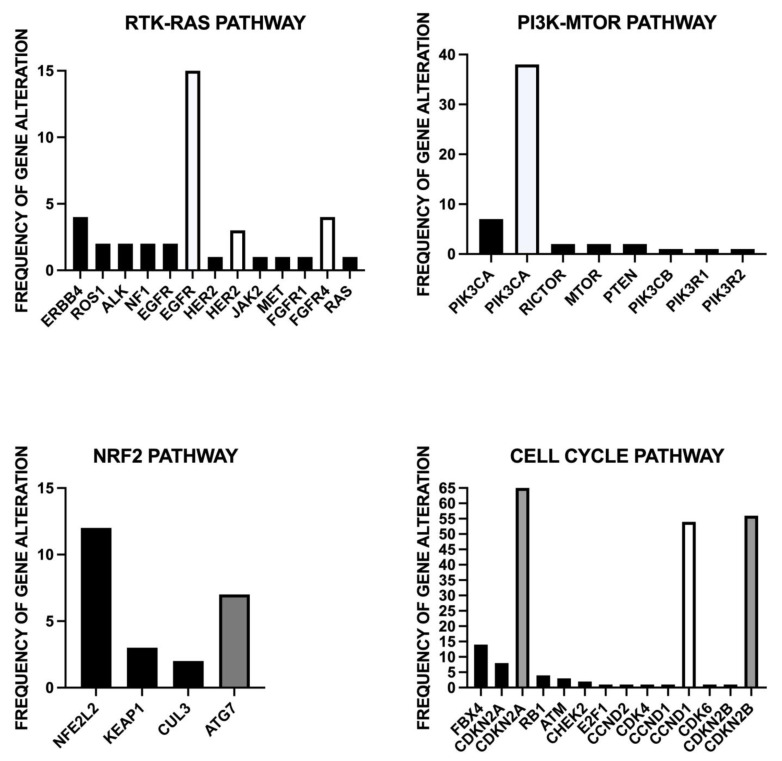
Main genomic alterations of four pathways, RTK-RAS, PI3K-MTOR, NRF2 and cell cycle, which can be targeted at the therapeutic level.

As discussed above, about 20% of ESCCs display mutations of the genes pertaining to the RTK-RAS pathway, and several of these genes are druggable, such as *EGFR, HER2*, *RPS1, ALK, MET, FGFR1* and *KRAS* [32] (Figure 1).

#### 4.2.1. EGFR

*EGFR* is mutated in about 2% of ESCCs [32]. Furthermore, the *EGFR* gene in amplified in about 10–20% of ESCCs; *EGFR* amplification is associated with EGFR overexpression [58]; *EGFR* amplification if a negative predictor of survival, as suggested by the observatio n that patients with *EGFR* overexpression exhibit a shorter survival rate compared with those with a lower EGFR expression [59].

Studies carried out in other solid tumors have shown that *EGFR* alterations can be targeted by specific pharmacologic agents. Preclinical studies using various agents targeting EGFR, such as gefitinib, lapatinib and anti-EGFR monoclonal antibodies (cetuximab or mimotuzumab), have shown significant antitumor activity against WSCC tumor cell overexpression in EGFR. Various clinical trials have evaluated the safety and the therapeutic impact of small molecule inhibitors or of monoclonal antibodies targeting EGFR for the treatment of ESCC patients. Two phase I/II studies evaluated the combination of chemoradiotherapy with the small molecule inhibitor erlotinib; in one of these two studies, in 21 ESCC-treated patients, 38% CE, 47% PR and 14% SD were observed [60,61]. In a randomized phase III trial, Xie et al. evaluated the effect of the addition of erlotinib, an EGFR inhibitor, to chemoradiotherapy in ESCC patients with locally advanced disease; erlotinib improved the median overall survival rate compared with chemoradiotherapy alone (39.4 months vs. 27.4 months, respectively) [62]. Two other studies evaluated the EGFR inhibitor icotinib [63,64]. In a single-arm phase II clinical trial, icotinib monotherapy elicited a 16.7% ORR and 46.3% DCR in pretreated ESCC patients with advanced disease [63]. In a randomized phase II clinical study, icotinib plus radiotherapy was compared with radiotherapy alone in older ESCC patients with unresectable tumors: icotinib plus radiotherapy improved the median overall survival compared with radiotherapy alone [64].

An AIO/EORTC POWER phase III trial randomized 146 ESCC patients with advanced disease to receive chemotherapy (cisplatin plus 5-fluorouracil) alone or chemotherapy plus panitumumab (an anti-EGFR mAb); however, the addition of panitumumab did not improve survival, and the study was stopped early due to a higher mortality rate in the panitumumab-treated group [65].

Lu et al. recently reported the results of a randomized phase II clinical study evaluating the effect of adding cetuximab to paclitaxel–cisplatin chemotherapy compared with chemotherapy alone in ESCC patients with metastatic disease not previously treated; in the whole population of treated ESCC patients, the mPFS was 5.7 months for cetuximab + chemotherapy compared with 4.2 months for chemotherapy alone; in the selected population of ESCC patients with EGFR amplification, the mPFS was 5.45 months for cetuximab + chemotherapy compared with 2.99 months for chemotherapy alone [66]. However, the overall survival was not significantly different in the two treatment arms in the whole ESCC population (11.5 months in the chemotherapy + cetuximab group compared with 10.5 months in the chemotherapy alone group); however, a clear trend of overall survival was observed considering only the EGFR-amplified patients (17.1 months versus 6 months in the cetuximab + chemotherapy and chemotherapy alone, respectively) [66].

In conclusion, additional studies will be required to prove a real benefit related to the administration of drugs targeting EGFR in ESCC patients with advanced or metastatic disease.

#### 4.2.2. FGFR

The FGF pathway is altered in some ESCC patients, including *FGFR1* mutations (1%); *FGFR2* amplification (4%); and *FGF3, FGF4* and *FGF19* (each > 50%). However, the biological role of FGF and its receptors in ESCC remains unclear. The FGFR4 inhibitor H3B-6527 induces an inhibition in the growth of ESCC both in vitro and in vivo through a blockage of the PI3K and MAPK signaling pathways [67]. Furthermore, the FGFR inhibitor AZD 4245 can increase the sensitivity of ESCC with elevated levels of activated FGFR1 to gefitinib, thus suggesting a potential treatment strategy [68]. FGF-2, by binding to FGFR 1–4, mediates the activation of numerous cell signaling pathways, including MAPK/ERK and PI3K-AKT-mTOR, thus sustaining survival and promoting the proliferation of ESCC [69]. FGF-2 overexpression is associated with an increased risk of recurrence and poor survival in esophageal cancer [70].

Other studies have shown that FGFR2-induced cell signaling, with AKT activation, is a driver of keratinocyte differentiation and regulates cell differentiation in ESCC [71] and the survival/maintenance of cancer stem-like cells in ESCC [69]. These observations suggest a possible role for FGFR2 inhibitors in reducing the survival of ESCC cancer stem cells.

Other studies have explored the expression of FGFR2 and its upstream regulator *miR-671-5p* in ESCC, showing an association between higher levels of FGFR2 and lower levels of *miR-671-5p*; high levels of FGFR2 induce the progression of ESCC due to the activation of the ERK and AKT pathways, while high levels of *miR-671-5p* induce the reduced expression of FGFR2 and the inhibition of ESCC progression [72].

Currently, few clinical trials with FGFR inhibitors in EC patients are ongoing, such as NCT 026,996,606, involving the FGFR inhibitor edafitinib, and NCT 01,795,768, involving the FGFR inhibitor AZD4547.

#### 4.2.3. HER2

*HER2* gene alterations, both mutations and gene amplifications, are markedly less frequent in ESCC than in EAC, thus indicating that *HER2* targeting is a potential therapy only in a minority of ESCC patients. Thus, Rong et al., in a retrospective analysis of 857 consecutive ESCC patients, showed that with immunohistochemistry only 1.5% of cases displayed strong HER2 positivity (3+) and 6.1% showed equivocal HER2 positivity (2+): dual-color in situ hybridization showed that 100% of patients with HER2 (3+) exhibited *HER2* gene amplification, whereas only 18.5% of those with HER2 (2+) had *HER2* gene amplification [73]. Therefore, 1.5% of ESCC patients overexpressed HER2 protein, and about 3% displayed *HER2* gene amplification [72]. *HER2*-overexpressing ESCCs do not differ from the other ESCC tumors for cell differentiation stages [73].

Another study based on a meta-analysis involving 1515 patients showed a higher rate of HER2 overexpression/amplification, which is estimated to occur in 8.6% of ESCC patients [74].

Currently, *HER2*-directed therapy is not used in ESCC, and there are no published studies reporting on HER2-targeted therapy in HER2-positive ESCC patients. The ongoing clinical trial HERES NCT 05,170,256 is evaluating trastuzumab (an anti-HER2 monoclonal antibody) and standard treatment with chemo- and immunotherapy as a first-line treatment for HER2-positive ESCC patients.

### 4.3. PI3K/AKT/mTOR

Mutations of the genes involved in the PI3K/AKT/mTOR signaling pathways are observed in about 15% of all ESCCs [32]. The most frequent alterations occur at the level of the *PI3KCA* gene. Shigaki et al., using whole genome sequencing, reported *PIK3CA* mutations at the level of exon 9 or exon 20 in 21% of ESCC patients [75]. Chang et al. reported an amplification of the *PIK3CA* gene in 38.3% of ESCC patients; however, cooccurring gene amplification and gene mutations are rarely observed in ESCC patients [31]. Compared to normal esophageal tissue, PIK3CA was significantly overexpressed in ESCC cancer tissue and its overexpression wasn indepenedently associated with higher risk of local recurremce [76]. *PIK3CA* mutations are more frequent in ESCC than in EAC [77]; interestingly, *PIK3CA* mutations are also frequent in other squamous tumors, such as lung and head and neck tumors [77]. Hot spot *PIK3CA* mutations (1624: pGlu542Lys; 1633: Glu545Lys) are enriched in ESCC with the APOBEC mutational signature [77]. Kim et al. reported *PIK3CA* amplification in 10.5% of ESCC patients undergoing curative esophageal resection; patients with *PIK3CA* amplification displayed a significantly shorter disease-free survival rate compared with those without this amplification [78].

Mutations of other components of these pathways, including *RICTOR, MTOR, PTEN, PiK3CB, PIK3R1, PIK3R2, AKT1, AKT2* and *AKT3* are less frequently observed than *PIK3CA* mutations [32].

A recent study reported the first clinical results using BKM 120—an oral pan class I PI3K inhibitor with promising activity in several cancers—as a form of monotherapy in a cohort of 42 patients with pretreated advanced ESCC; of the 42 patients enrolled, 20 had stable disease and 2 had confirmed partial responses [79]. In total, 23% of these patients had genetic alterations in PI3K pathways; of the two patients with partial responses, one displayed *PI3KCA* amplification and the other one had a *PI3KCA* mutation [79]. These observations support further clinical explorations of PI3K inhibitors in ESCC patients.

### 4.4. KEAP1-NRF2

Nuclear factor erythroid 2-related factor 2 (NFE2L2 or NRF2) is a master transcription factor that controls the expression of many cytoprotective genes. The expression of NFE2L2 at the protein level is controlled by proteasomal degradation mediated by two E3 ubiquitin ligase complexes, kelch-like ECH-associated protein 1 (KEAP1)-Cullin 3 (CUL3) and beta-transducin repeat-containing ligase (beta TrCP)-Cullin1 (CUL1) [80]. In basic conditions, NFE2L2 protein levels are low as a result of degradation enzymes; however, stress conditions mediated by reactive oxygen species or toxic chemicals induce an increase in NFE2L2 protein levels through the attenuation of the ubiquitin ligase activity of the two degradation complexes. Once activated, NFE2L2 translocates to the nucleus where it activates a set of cytoprotective genes. NFE2L2 protein levels may also be increased by somatic mutations of *NFE2L2* altering the sensitivity of the NFE2L2 protein to the degradation enzymes (most *NFE2L2* mutations occur in KEAP1-interacting domains) or by inactivating mutations of the components of the degradation complexes, such as *KEAP1* mutations.

NFE2L2 plays an important role in esophagus physiology, affecting development, differentiation and metabolism. Interestingly, NFE2L2, both at the mRNA and protein level, is expressed in normal esophageal tissue at higher levels compared with other tissues [80].

Mutations of the *NFE2L2* gene are observed in 10–20% of ESCC cases; other genes of the *NFE2L2* pathway are also mutated in ESCC: somatic mutations of *KEAP1* (2–4% of cases), somatic mutations or deletions of *CUL3* (1–5%) and deletions of *ATG7* (6% of cases) [27,81]. The frequency of NFE2L2-activated ESCCs is higher than that of ESCCs bearing alterations in the NEFE2L2 system. NFE2L2 confers chemoradiotherapy resistance and a poor prognosis. Several studies support the conclusion that NRF2 expression in ESCC is associated with resistance to chemoradiation therapy. Thus, Kawasaki et al. explored 46 patients with ESCC who received curative surgery after chemoradiation therapy; 39% of these patients displayed NFE2L2 expression, associated with an unfavorable response to chemoradiation therapy and with lymph node metastases [82]. Wang et al. explored 164 cases of ESCC with advanced disease undergoing treatment with chemoradiotherapy and showed that patients with high nuclear NFE2L2 expression exhibited lower ORR, PFS and OS compared with those with a low nuclear NFE2L2 expression [83]. In chemoradioresistant patients, NFE2L2 expression positively correlates with p-p62 expression [83]. Hsieh et al. explored 164 ESCC patients with advanced ESCC undergoing treatment with radiotherapy and identified a gene expression signature associated with NRF2 activation in poor responder patients [84]. Furthermore, an inverse relationship between NFE2L2 levels and SOX17 levels was observed: SOX17 acted as a transcriptional repressor of NFE2L2, and in some ESCCs, SOX17 expression was low due to promoter hypermethylation [84]. In particular, patients with low nuclear SOX17 expression displayed poor responses to chemoradiotherapy [84].

Jiang et al. explored the expression and prognostic impact of NFE2L2 in ESCC patients. An immunohistochemical analysis carried out on 130 primary ESCCs showed that 59% of these tumors displayed NFE2L2 expression; among the positive cases, NFE2L2 expression was localized in the cytoplasm in 60% of cases; in the nucleus in 18% of cases; and both in the cytoplasm and the nucleus in 22% of cases [85]. In normal esophageal tissue, NFE2L2 protein is predominantly localized in the cytoplasm. ESCCs positive for NFE2L2 tend to also be positive for NAD(P)H quinone oxidoreductase (NQO1), a downstream target of NFE2L2, and there is a positive correlation between these two proteins. Interestingly, NQO1 positivity has been observed to be higher in ESCC samples with a nuclear NFE2L2 expression [85]. *NFE2L2* alterations were observed in about 53 ESCCs with double the NFE2L2 and NQO1 expression. High nuclear NFE2L2 expression predicted shorter overall survival in patients with the dual expression of NFE2L2 and NQO1; furthermore, the presence of both nuclear NFE2L2 expression and the presence of *NFE2L2* alterations were shown to accurately predict the prognosis of ESCC patients [85].

The mechanisms through which alterations of NRF2 pathway genes favor ESCC development are complex and have been, in part, elucidated through the study of experimental mouse models. To this end, it was fundamental to develop in vivo models in which *NRF2*-deleted cells or *NRF2*-activated cells coexist with *NRF2*-normal cells. A study on a model of coexistence for *NRF2*-deleted and *NRF2*-normal cells in mouse esophagi showed that, while *NRF2*-deleted cells were maintained in the esophageal epithelium under normal conditions, these cells were selectively eliminated from the epithelium following exposure to chemical carcinogens, and this may be due to a mechanism of competition between deleted and normal *NRF2* cells [86]. This conclusion was also supported by a study on a model of the coexistence of *KEAP1*-deleted/*NRF2*-activated and *KEAP1*-normal/*NRF2*-normal cells; in this model, constitutive *NRF2* activation in a part of the cells promotes the selective elimination of *NRF2*-activated cells and the accelerated proliferation of neighboring *NRF2* normal cells [87]. This mechanism of cell competition induces DNA damage in neighboring *KEAP1*-normal/*NRF1*-normal cells, a process that predisposes these cells to chemically induced ESCC [87].

It is not easy to explain, according to the cell-active competition model, the development of NRF2-activated ESCCs. To explain this process, it is of fundamental importance to consider that *NRF2* mutations—a late event during ESCC development [42]—act together with many other gene alterations, including *TP53* mutations. The mutations of these genes, which initiate the tumorigenic process, cooperate with *NRF2* mutations and allow for the expansion of *NRF2* mutant subclones during tumor evolution.

The development of NRF2 inhibitors was considered a rational approach for the treatment of NRF2-activated cancers, including ESCC. Several compounds inhibiting NRF2 activity were recently developed. ML385, one of these inhibitors, was identified through high-throughput screening [88]. This inhibitor targets NRF2 DNA binding and blocks NRF2 transcriptional activity [88]. MLN385 exerts inhibitory activity on *KEAP1*-mutated non-small-cell lung cancer cells and is a candidate drug for the treatment of cancers with NRF2 activation, including ESCC.

Other recent studies support the possible therapeutic inhibition of tumors exhibiting NRF2 activation using glutaminase inhibitors. In fact, one study showed that hyperactive NRF2 causes metabolic reprogramming of the mouse esophagus through the transcriptional regulation of some metabolic genes, inducing enhanced activity in the glycolytic pathway; blocking glycolysis transiently inhibits the proliferation of human ESCC cells [89]. A second study showed that lung adenocarcinomas with *KEAP1* mutations and consequent NRF2 activation exhibit increased expression in genes involved in glutamine metabolism and enhanced glutamine dependence, which can be therapeutically targeted using the glutaminase inhibitor telaglenastat [90].

Another study showed that *KEAP1/NFE2L2* mutations predict lung cancer radiation resistance, which can be targeted by glutaminase inhibition: glutaminase inhibitors preferentially radio-sensitize *KEAP1/NFE2L2*-mutant tumor cells via the depletion of glutathione and increased radiation-induced DNA damage [91].

### 4.5. CDK4/CDK6 Targeting

Abnormalities of cell cycle regulators are frequent in ESCC. In particular, *Fbxo4* loss and *cyclin D1* amplification occur frequently in ESCC and are involved in disease development and progression. The dysregulation of the Fbxo4-cyclin D1 axis determines abnormalities in Rb and mTORC1 expression, inducing metabolic reprogramming and glutamine activation. This metabolic reprogramming causes reduced energy production and the increased sensitivity of ESCC cells to combination treatments with glutaminase inhibitor plus metformin [92]. Importantly, CDK/6 inhibition enhances the efficiency of EGFR inhibition [93]; enhances radiosensitization by activating autophagy signaling via mTOR suppression [94]; and induces apoptosis and senescence in ESCC cells [95].

## 5. Immunotherapy of ESCC with Immune Checkpoint Inhibitors

The discovery of immune checkpoint (IC) proteins represents a major breakthrough in tumor immunotherapy; IC proteins act as potent suppressors of the immune system through multiple mechanisms, and their inhibition represents a potent and unique tool to stimulate antitumor immune responses. Thus, immune checkpoint inhibitors (ICIs) represent a leading approach in tumor immunotherapy; these inhibitors have been used for the treatment of many tumors and have good tolerability, achieving, in some cases, stable responses even in patients with metastatic and chemotherapy-resistant disease.

### 5.1. Advanced/Metastatic Setting, Second-Line

Many studies carried out in the last few years support the idea that there are clinical benefits that can be derived from ICI administration in ESCC patients with advanced/metastatic disease.

The phase Ib KEYNOTE-028 trial involved the treatment of 23 patients with previously treated advanced EC (78% with ESCC) with the anti-PD1 monoclonal antibody pembrolizumab. An ORR of 30% was observed with a PFS of 1.9 months, and an overall survival of rate of 7 months was observed, with a PFS of 1.9 months and OS of 7 months [96]. A KEYNOTE-181 phase III randomized trial compared pembrolizumab plus chemotherapy to chemotherapy alone in 628 patients with previously treated advanced EC (63% with ESCC), showing that ESCC patients (with a PD-L1 CPS of ≥ 10) had an OS of 43% after 12 months with pembrolizumab plus chemotherapy compared with 20% with chemotherapy alone and a median OS of 8.2 months using pembrolizumab plus chemotherapy compared with 7.1 months with chemotherapy alone [97]. A subgroup analysis of Asian patients enrolled in this trial showed an OS of 10 months in patients treated with pembrolizumab plus chemotherapy compared with 6.5 months in those treated with chemotherapy alone [98]. An analysis of clinical responses on the function of the tumor with respect to PD-L1 positivity showed that a PD-L1 CPS of ≥ 1 is an appropriate cutoff and predictive biomarker of pembrolizumab efficacy [99].

The phase II randomized ESCORT trial evaluated the anti-PD1 mAb camrelizumab in 428 previously treated ESCC patients with advanced disease: 228 patients received camrelizumab, and 220 patients were treated with chemotherapy. The PFS was 1.9 months in both treatment groups, while the OS was 8.3 months for the camrelizumab group compared with 6.2 months for the chemotherapy group [98].

Another anti-PD1 monoclonal antibody, nivolumab, was assessed in the ATTRACTION-1 phase II trial in 65 ESCC patients who were refractory to platinum, taxane and fluoropyrimide chemotherapy. Nivolumab decreased the tumor load and target tumor lesions in 45% of the treated patients; the median PFS was 1.5 months and the median OS was 10.8 months [100]. The randomized phase III ATTRACTION-3 trial involved the treatment of 417 ESCC patients with advanced disease with either nivolumab or chemotherapy; nivolumab elicited a significant improvement in the OS, with a median OS of 10.9 months compared with 8.4 months for the patients treated with chemotherapy [101].

No direct comparisons of ICIs in a second-line setting for ESCC exist, and only indirect comparisons are available. Zhou et al. attempted a comparative analysis of the results of the three randomized studies (KEYNOTE-181, ATTRACTION-3 and ESCORT), comparing patients with advanced ESCC in the second-line setting treated with three different anti-PD1 inhibitors (pembrolizumab, nivolumab and camrelizumab) or with chemotherapy alone [102]. This analysis showed an indirect similar survival benefit for nivolumab, pembrolizumab and camrelizumab; nivolumab was better for patients with poorer performance status; camrelizumab and pembrolizumab displayed better PFS and ORR values [102]. This indirect comparison had many intrinsic limitations due to the heterogeneity of the patients enrolled in these three different studies.

Other recent studies have explored different ICIs in second-line treatments of ESCC patients. The phase III RATIONALE-302 trial randomized 512 ESCC patients, previously treated with either tislelizumab (anti-PD1) or chemotherapy (docetaxel, paclitaxel or irinotecan) as second-line treatments. The results of this study showed an improvement in the median OS among patients treated with tislelizumab for 6.3 months compared with patients treated with chemotherapy for 8.6 months [103]. A clinical benefit was observed in all subgroups defined according to a PD-L1 positivity score [103]. Furthermore, tislelizumab treatment was associated with a more durable antitumor response (7.1 months vs. 4.0 months) [103].

The randomized, multicenter phase II trial ORIENT-2 evaluated sintilimab, a PD1 inhibitor, versus chemotherapy in 181 ESCC patients with advanced disease as second-line therapy; the median OS in the sintilimab group was significantly improved compared with the chemotherapy group (7.2 months vs. 6.2 months); furthermore, sintilimab increased the ORR (12.6% vs. 6.3%) and the duration of the response (8.3 months vs. 6.2 months) [104]. Interestingly, after the initial disease progression, 37 patients in the sintilimab group continued to receive the ICI treatment, and 28 patients discontinued this treatment; a post hoc analysis of these patients showed a median OS for patients with continuous sintilimab treatment, markedly improved compared with those who discontinued this treatment [104]. An analysis of biomarkers associated with responses to sintilimab treatments showed that patients with a neutrophil-to-lymphocyte ratio (NLR) that was low (<3) displayed a better OS compared with those with a high NLR (>3); patients with high T cell receptor clonality and a low tumor-burden index had the highest OS and PFS values [104].

The phase II RAMONA trial enrolled 66 older ESCC patients with recurrent disease in second-line therapy: 67% of these patients received nivolumab and ipilimumab (anti-CTL-A4), and received 33% nivolumab alone [105]. Median overall survival was 7.2 months, which was significantly higher than what has been historically observed for patients receiving standard chemotherapy [105].

A recent phase II study reported the preliminary results of a new drug combination based on a PD1 inhibitor (camrelizumab) with an anti-angiogenesis inhibitor (apatinib) and a DNA topoisomerase I inhibitor (irinotecan) used as second-line treatments in an initial group of 16 ESCC patients with advanced ESCC: 1% CR, 8% PR and 4% SD were observed in these patients [106]. These preliminary results are promising, but additional data on the 42 patients enrolled in a phase II study and a randomized phase III trial are needed to assess the real therapeutic impact of this triplet regimen in second-line ESCC patients.

SHR-1210 is a new, fully humanized anti-PD1 monoclonal antibody that was tested in a monotherapy in 30 ESCC patients with advanced/metastatic disease as a second-line therapy, achieving 33% objective responses: objective responses were more common among patients with PD-L1-positive tumors with > 5% positive cells and high tumor mutational burden [107].

### 5.2. Advanced/Metastatic Setting, First-Line

Several studies have explored the therapeutic efficacy of ICIs administered in combination with standard chemotherapy as a first-line treatment in ESCC patients.

The phase III Keynote-590 trial involved the enrollment of 749 patients with metastatic ECs (73% ESCC) who were randomized to receive standard first-line chemotherapy (cisplatin and 5-fluorouracil) alone or in combination with pembrolizumab [108]. The addition of pembrolizumab to chemotherapy improved the OS to about 5 months in ESCC patients with PD-L1 CPS ≥ 0 and 3 months in the whole ESCC population [108]. The combined treatment had a manageable safety profile. A recent update of the results observed in this study, with a longer follow-up, showed that 20% of patients in the pembrolizumab arm maintained a response compared with 6% among the patients treated with chemotherapy alone [109].

The ESCORT-1 phase III clinical trial randomized 595 Chinese patients with untreated metastatic ESCC to receive either standard chemotherapy treatments (cisplatin and paclitaxel) or chemotherapy in combination with camrelizumab [110]. The addition of camrelizumab to chemotherapy improved both the OS (15.3 vs. 12.0 months) and the PFS (6.9 vs. 5.6 months) [110].

The phase III CHECKMATE 648 trial randomized 970 untreated ESCC patients with advanced disease to receive nivolumab plus chemotherapy (cisplatin and 5-fluorouracil; nivolumab and ipilimumab; or chemotherapy alone, independently of the PD-L1 expression). These three arms of treatment displayed mOS values of 13.2, 12.7 and 10.7 months; at the level of patients with PD-L1 ≥ 1%, the mOS values in the three groups were 15.4, 13.7 and 9.1 months, respectively [111]. The ORRs in the three arms were 53%, 35% and 20%, respectively [111].

The phase III ORIENT-15 trial randomized 659 untreated ESCC patients with advanced disease to receive sintilimab plus chemotherapy (cisplatin, paclitaxel and 5-fluorouracil) or chemotherapy alone. Patients treated with sintilimab and chemotherapy had a better OS (16.7 vs. 12.5 months) and PFS (7.2 vs. 5.7 months) in the whole population; in patients with PD-L1 CPS ≥ 10, the mOS was 17.2 months for sintilimab plus chemotherapy compared with 13.6 months for chemotherapy alone [112]. The phase III randomized JUPITER-6 trial evaluated the efficacy of toripilimab in association with standard chemotherapy (cisplatin and paclitaxel) compared to chemotherapy plus placebo in a group of 514 Asian untreated ESCC patients. The addition of toripilimab to chemotherapy significantly improved the OS compared with chemotherapy alone (17 vs. 11 months, respectively) [113]. Furthermore, toripilimab also significantly improved PFS (HR: 0.58, *p* > 0.0001) [113].

Although several anti-pD-L1 antibodies have provided benefits in combination with chemotherapy for patients with other solid tumors, no report has explored the use of these antibodies in ESCC patients. However, a recent study by Mu et al. reported the results of a phase II multicenter, open-label trial evaluating the efficacy and safety of anti-PD-L1 monoclonal antibody SHR-1316 administered in combination with liposomal irinotecan and 5-fluorouracil to 23 patients with advanced ESCC as a first-line treatment. The median OS was 11.6 months, the median PFS was 8.5 months, and the ORR and DCR were 52.2% and 73.9%, respectively [114]. Thus, this therapeutic regimen seemed to have promising efficacy and a manageable safety profile in ESCC patients as a first-line treatment.

### 5.3. ICIs in Association with Neoadjuvant Chemoradiotherapy in Patients with Locally Advanced ESCC

Neoadjuvant chemoradiotherapy (nCRT) is the standard of treatment for ESCC patients with locally advanced disease; however, the outcomes of these patients are not optimal, with a five-year survival ranging from 25% to 50% and relapse recurrence rates ranging from 30% to 50%. Recent studies suggest that the combination of ICIs with nCRT could represent a new strategy to improve the efficacy of neoadjuvant chemoradiation treatments. In this context, two different types of studies can be distinguished: (i) ICIs for locally advanced resectable ESCCs, subdivided into those in which ICIs are combined with nCRT and those in which ICIs are combined with neoadjuvant chemotherapy (nC), and (ii) ICIs for locally advanced unresectable ESCCs, subdivided into those combined with definitive (dRT) and those combined with definitive chemoradiotherapy (dCRT).

#### 5.3.1. Immunotherapy Studies in ESCC Patients with Resectable Tumors

Several phase I and II clinical studies have explored the safety and efficacy of ICIs added to nCRT in ESC patients with locally advanced ESCC. It is important to point out that phase II randomized clinical trials have shown that, in ESCC patients with resectable disease, nCRT before surgery improves the OS (100.1 months vs. 66.5 months) and DFS (100.1 months vs. 41.7 months) compared with surgery alone [115]. In a group of patients treated with nCRT, the pathologic complete response rate (pCRR) was 43.2% [115].

In a single-arm phase II clinical trial, Hong et al. reported on 28 ESCC patients treated with pembrolizumab plus chemotherapy before surgery and with pembrolizumab after surgery with a pCRR of 46.1% and 1-year OS of 89.3% [116].

The phase I PALACE-1 trial evaluated the safety and efficacy of an nCRT regimen including the addition of pembrolizumab in 20 ESCC patients: in 18 patients undergoing surgical resection, the pCRR was 55.6% [117]. A phase III (PALACE-2) study will be performed to confirm the safety profile and to demonstrate the efficacy of this nCRT regimen [118].

Two recent studies presented at the ASCO 2022 Meeting reported the association of toripilimab with nCRT (54% of pCRR) [119] or radiotherapy (55% of pCRR) [120].

Since CRT is associated with a higher pretreatment mortality rate compared with surgery alone (2.2% vs. 0.4%), some studies have explored the efficacy of adding ICIs to neoadjuvant chemotherapy alone without radiotherapy. Thus, several studies have explored the association between ICIs and nC.

A prospective study involving a neoadjuvant regimen based on camrelizumab, paclitaxel and carboplatin was administered to 16 ESCC patients, achieving a pCRR of 31.3%; at a one-year follow-up, the OS and PFS were 90.9% and 83%, respectively [121].

Yang et al. reported results on 23 ESCC patients with stage II or III resectable ESCC undergoing neoadjuvant treatment with camrelizumab, nab-paclitaxel and carboplatin before surgery: 25% of the patients had a pCRR, and 50% had a major pathological response; the patients with a high tumor mutation burden and high expression of PD-L1 were more frequently in the pCRR group than the non-pCRR group [122].

The NICE study was a prospective, multicenter, open, single-arm phase II trial in which ESCC patients with locally advanced resectable disease received a neoadjuvant treatment (NICE regimen) with camrelizumab, albumin paclitaxel and carboplatin for two cycles before surgery; pCR was identified in 42.5% of the patients, and 10.6% of the patients had pPCR in the primary tumor but residual disease in lymph nodes alone [123]. To demonstrate an improvement in survival elicited by the addition of ICI to the neoadjuvant chemotherapy regimen, a controlled phase III trial was designed [124].

FRONTIER was a phase I trial aiming to evaluate the safety and efficacy of nivolumab administered in association with two different chemotherapy regimens [125]. In cohort A, the patients received nivolumab plus chemotherapy (5 fluorouracil + paclitaxel), and in cohort B, the patients received the same treatment as in A, preceded by a nivolumab infusion: 33% of the patients in cohort A achieved a pCR [126]. In cohort C, the patients received nivolumab plus chemotherapy (5-fluorouracil + docetaxel), while in cohort D, the patients received the same treatment as in C, preceded by a nivolumab infusion: 16.7% and 50% of the patients achieved a pCR in cohorts C and D, respectively [127].

The phase II KEYSTONE-001 prospective, single-center study investigated pembrolizumab in combination with nC in 43 ESCC patients with stage III, locally advanced tumors and reported a pCRR of 41.4%, with 72.4% of patients achieving a major pathological response [128]. According to the results of this trial, a phase III trial (KEYSTONE-002) was proposed, aiming to compare the efficacy and safety of pembrolizumab combined with nC versus nCRT, followed by surgery for locally advanced ESCCs [129].

Although most studies on the association between ICIs within an nC or nCRT regimen have involved concurrent treatments with ICIs and chemotherapeutic agents, chemotherapy drugs administered concurrently with ICIs could kill T cells activated by anti-PD1 or anti-PD-L1 antibodies and, thus, could inhibit the effects of ICIs. Xing et al. addressed the important problem of the optimal timing of the administration of ICIs within the context of nC in a phase II study in which toripalimab was administered to some patients contemporaneously with nC (cisplatin and paclitaxel) and to other patients 3 days after nC [130]. Administering toripalimab 3 days after administering chemotherapy resulted in a higher pCRR than concurrent administration [130].

The risk of disease recurrence remains high for patients who have residual disease after nCRT and surgery. Therefore, there is a strong rationale to evaluate adjuvant treatments after surgery to attempt to decrease the risk of disease recurrence. In this context, the randomized phase III CheckMate 577 study investigated 1085 patients with EC or gastroesophageal junction cancer who did not achieve pCR after nCRT; these patients were randomized to receive nivolumab for 1 year or placebo [131]. The median DFS was 22.4 months for patients receiving nivolumab and 11.0 months for the placebo group; the median-distance metastasis-free survival rate was 28.3 months for the nivolumab group compared with 17.6 months for the placebo group [131]. This improvement in survival observed for patients treated with nivolumab was particularly evident for the ESCC patients enrolled in this study [131]. The frequency of grade 3–4 adverse events was higher in the nivolumab group compared with the placebo group (13% vs. 6%, respectively) [131].

However, a phase II, placebo-controlled, randomized study carried out on 86 ESCC patients undergoing nCRT and treated with adjuvant durvalumab or placebo failed to show any benefit at the level of DFS or OS [132]. It is important to note, however, that this study, contrary to the other study, enrolled both postoperative pCR and non-pCR patients. This apparent discrepancy between these two studies strongly suggests that non-PCR patients mostly benefit from postoperative adjuvant ICI treatments.

#### 5.3.2. Immunotherapy Studies in ESCC Patients with Unresectable Tumors

Patients with locally advanced unresectable ESCC who cannot tolerate treatment with CRT are treated with definitive radiotherapy. Some recent phase I/II studies have evaluated the safety and feasibility of radiotherapy plus ICIs for locally advanced ESCC. A phase II trial reported the results observed in 14 ESCC patients with locally advanced unresectable ESCC undergoing treatment with radiotherapy and camrelizumab: the treatment was well tolerated (42.9% of patients experienced grade 1–2 adverse events) and 1 patient showed a complete response, while the remaining 13 patients displayed a partial response [133]. Another phase Ib trial explored radiotherapy plus an anti-PD1 antibody (camrelizumab) as a first-line therapy for patients with unresectable locally advanced ESCC; the patients were treated with 54–60 Gy radiation [134]. The median OS and PFS were 16.7 months and 11.7 months, respectively [134]. Some biomarkers related to PD-L1 expression and some T lymphocyte subsets were associated with clinical responses [134].

A recent phase II study explored the safety and efficacy of adding durvalumab (anti-PD1) and tremelimumab (anti-CTLA-4) to definitive CRT in 40 patients with locally advanced unresectable ESCC; after completing CRT with immunotherapy, the patients received two cycles of treatment with durvalumab and tremelimumab, followed by durvalumab monotherapy for up to two years after enrolment [135]. After 24 months of follow-up, the PFS and OS were 57.5% and 75%, respectively; patients with PD-L1-positive tumors had higher PFS and OS values than those patients with PD-L1-negative tumors [135]. In a historical control group, no differences were observed between PD-L1-positive and PD-L1-negative tumors [135].

A phase Ib trial explored the safety and feasibility of combining concurrent CRT with camrelizumab as a first-line treatment for patients with locally advanced ESCC. The study enrolled a total of 20 patients. The most common treatment-related grade 3 adverse events were radiation esophagitis (20%) and esophageal fistulas (10%); 65% of patients exhibited an objective response. At a median follow-up of 23.7 months, the OS and PFS ranged from 8.2 to 28.5 months and 4.0 to 28.5 months, respectively; the 12-month and 24-month OS rates were 85% and 69.6%; the PFS rates were 80.0% and 65%; and the tumor PD-L1 expression was associated with OS [136].

Wei et al. performed a study to evaluate the efficacy of PD1 inhibitors in combination with concurrent CRT/C for 96 patients with unresectable ESCC in real-world conditions [137]. As compared with a control group, the PFS and OS were significantly higher in the ICIs group [137].

In conclusion, the studies carried out up to now on unresectable ESCC patients with locally advanced disease support the safety and feasibility of combining RT or CRT and immunotherapy.

Ongoing phase III randomized multicenter trials are evaluating the real therapeutic impact of CRT in ESCC patients with unresectable, locally advanced disease.

## 6. Conclusions

The data analyzed in this review on ESCC highlight some important findings that can be summarized as follows:

A better definition of molecular abnormalities observed in ESCC and their intertumoral and intratumoral heterogeneity is fundamental not only in understanding the molecular mechanisms underlying cancer development but also as a fundamental tool for the identification of therapeutic targets.

These studies suggest that about 40% of these molecular abnormalities are actionable. However, the first studies on the molecular targeting of some of these molecular abnormalities showed only a limited therapeutic benefit, if any, and this finding is not surprising given the consistent heterogeneity of ESCCs.

Novel ESCC molecular targets need to be further defined but different strategies may be required to target the NRF2 pathway, PIK3CA inhibition and CDK4/6 inhibition.

The recent introduction of immune checkpoint inhibitors has revolutionized the therapeutic landscape of ESCC patients with advanced/metastatic disease, and the combination of an agent targeting PD1 or PD-L1 with chemotherapy is now the standard of care for these patients.

However, the benefit of ICIs is limited to a minority of patients, and robust biomarkers to identify responsive patients are needed, avoiding unnecessary side effects for patients not responding to ICIs and limiting the excessive cost related to the administration of these drugs.

ICIs may also improve the benefit deriving from neoadjuvant chemotherapy or chemoradiotherapy for the treatment of ESCC patients with locally advanced disease, but the results of ongoing phase III studies are needed to assess their real therapeutic impacts in this setting. Similarly, ICI administration could provide some benefit in an adjuvant setting in patients with locally advanced ESCC to prevent or delay relapses.

Studies on the immunotherapy of ESCC patients suggest a two-step strategy in the optimal development of new therapeutic approaches: firstly, the identification of new treatment regimens to define the most promising in terms of therapeutic impact; secondly, the definition of patients who are responsive to this treatment through the development of robust biomarkers.
